# Treatment of Cloth by Antiseptic Substances

**Published:** 1918-01

**Authors:** 


***Treatment of Cloth by Antiseptic Substances.*** By Miss Mary Davies, Bacteriologist under the Robert Walton Goelet Research Fund. Abs. of the portion of the article bearing upon the louse problem. Trans. and Abs. from the *Archives de Médecine et de Pharmacie Militaires*, Aug. 1916, t. LXVI, n° 2, p. 227. See also the *Lancet*, Sept. 30, 1916.

The author in collaboration with Kenneth Taylor proved *in vitro* that it is possible to render soldiers' clothing antiseptic for a period of over a month, so that it may not harbor infectious organisms or their spores. Clothing thus treated, even if exposed for long periods to bad weather and then contaminated with various bacilli, retards bacterial growth. The most effective antiseptic preparation for this purpose is a 5 o/o solution of pyxol. The author believes that underclothing thus treated would exert a sterilizing influence upon the skin.

She also believes, although she has made no experiments to confirm her opinion, that the diffusion of the antiseptic would be equally effective against lice. She concludes that the periodical treatment of soldiers' clothing by an antiseptic would more than compensate for the trouble or expense, because it would probably favor immunity against both bacteria and vermin. Since pyxol has given the best results in experiments, she thinks that in practice an antiseptic with cresol as a base would be the most satisfactory. It would be comparatively easy, she thinks, to apply the antiseptic in the rinsing water of the regular laundry establishments.

